# Construction of a Luminescent Cadmium-Based Metal–Organic Framework for Highly Selective Discrimination of Ferric Ions

**DOI:** 10.3390/molecules26226847

**Published:** 2021-11-13

**Authors:** Li-Li Xu, Qiu-Feng Zhang, Dong Wang, Guang-Wei Wu, Hong Cai

**Affiliations:** 1 School of Chemistry and Environmental Engineering, Hanshan Normal University, Chaozhou 521041, China; lilyncu@hstc.edu.cn (L.-L.X.); 2017137134@stu.hstc.edu.cn (Q.-F.Z.); wangd@hstc.edu.cn (D.W.); 2015136005@stu.hstc.edu.cn (G.-W.W.); 2 Guangdong Provincial Key Laboratory of Functional Substances in Medicinal Edible Resources and Healthcare Products, Chaozhou 521041, China

**Keywords:** metal–organic frameworks, luminescent, Fe^3+^ recognition, high selectivity

## Abstract

Fluorescent metal–organic frameworks (MOFs) are ideal materials for sensors because of their adjustable pore size and functional groups, which provide them with favorable metal ion selective recognition. In this paper, a new cadmium-based MOF was synthesized using Cd(NO_3_)_2_·4H_2_O and 3,3′,5,5′-biphenyltetracarboxylic acid by solvothermal method. CdBPTC owned three types of channels with dimensions of approximately 8.4 × 8.3 Å, 6.0 × 5.2 Å, 9.7 × 8.4 Å along a, b, and c axis, respectively. This MOF has high selectivity to ferric ions and shows excellent anti-inference ability toward many other cations. The results indicate that the fluorescence quenching efficiency of CdBPTC is close to 100% when the concentration of Fe^3+^ reaches 1.0 × 10^−3^ mol·L^−1^. Moreover, the luminescent intensity at 427 nm presents a linear relationship at a concentration range of 2.0 × 10^−4^~7.0 × 10^−4^ mol·L^−1^, which can be quantitatively expressed by the linear Stern–Volmer equation I_0_/I = 8489 [Fe^3+^] − 0.1400, which is comparable to the previously reported better-performing materials. Competitive energy absorption and ion exchange may be responsible for the variation in fluorescence intensity of CdBPTC in different Fe^3+^ concentrations.

## 1. Introduction

Iron ions play an irreplaceable role in living systems, and the lack or excess of ferrum in the human body causes serious dysfunction. Iron ions are an important component of hemoglobin and myoglobin but are also related to transferrin. In addition, the level of iron ions content has a profound impact on industrial and agricultural production, as well as daily life [1,2,3,4,5]. Therefore, the detection of iron ions is of great significance for environmental detection and life science. Currently, the methods for the determination of iron ions include oxidation–reduction titration, electrochemistry, inductively coupled plasma spectroscopy (ICP), inductively coupled plasma mass spectrometry (ICP–MS), flame atomic absorption spectrometry (FAAS), etc. Each approach has its advantages and disadvantages. For example, the methods of polarography and potassium dichromate titration analysis are simple to operate but with low sensitivity. Although FAAS and ICP–MS have good sensitivity, their high price, complicated pretreatment, and operation restrict their widespread use. In this context, the detection of iron ions based on luminescence has attracted much attention due to its simplicity, rapidity, and high sensitivity [6,7,8,9,10].

Metal–organic frameworks (MOFs) are new types of porous crystal materials [11,12] mainly composed of inorganic metal ions or metal clusters and organic ligands connected by coordination bonds to form regular two- or three-dimensional network structures, which have been widely applied in separation and storage of gases [13], chemical sensing [14], catalysis [15,16], food detection [17], biomedicine [18,19] and other fields [20,21]. Fluorescent MOFs and organic porous materials [22,23,24] are ideal candidates for sensors because of their adjustable pore size and functional groups, which provide them with favorable metal ion selective recognition. It has been reported in the literature that iron quenched the fluorescence of MOFs owing to the paramagnetic feature and intensely visible absorbance of Fe^3+^, thus forming a “turn-off” sensor. For instance, Lv. R. et al. [5] synthesized a new luminescent Cd-MOF, which can detect Al^3+^ and Fe^3+^ by means of fluorescence quenching. The detection limit reached 0.56 μM for Al^3+^ and 0.3 μM for Fe^3+^, respectively. Nevertheless, some examples selectively detected Fe^3+^ via the “turn-on” effect, such as Co-based JXUST-2 [25], and Tb-based Tb-TCPP [26,27], in which turn-on MOFs were used simultaneously as fluorescent sensors for Fe^3+^, Cr^3+^, and Al, and displayed a 10-fold fluorescence enhancement. However, few studies have used MOFs for the specificity recognition of Fe^3+^ through the turn-on or turn-off effect under different concentrations.

In this paper, a new cadmium-based MOF (named as CdBPTC) was synthesized using Cd(NO_3_)_2_·4H_2_O and 3,3′,5,5′-biphenyltetracarboxylic acid (H_4_BPTC) by solvothermal method and characterized by single-crystal X-ray diffraction (SXRD), powder X-ray diffraction (PXRD), thermogravimetric analysis (TGA), Fourier transform infrared spectroscopy (FTIR), and fluorescence spectroscopy. Fe^3+^ significantly affected the fluorescence properties of CdBPTC. Interestingly, the fluorescence intensity was higher than that of CdBPTC when the concentration of Fe^3+^ was in the range of 1 × 10^−9^~1 × 10^−5^ mol·L^−1^. Above 10^−4^ mol·L^−1^, the fluorescence intensity dropped sharply with turn-off effect, showing a linear relationship in the range of 2 × 10^−4^~7 × 10^−4^ mol·L^−1^.

## 2. Results and Discussion

### 2.1. Crystal Structure Description of {[Me_2_NH_2_]_2_[CdBPTC]·6H_2_O}n (CdBPTC)

**Figure 1:**
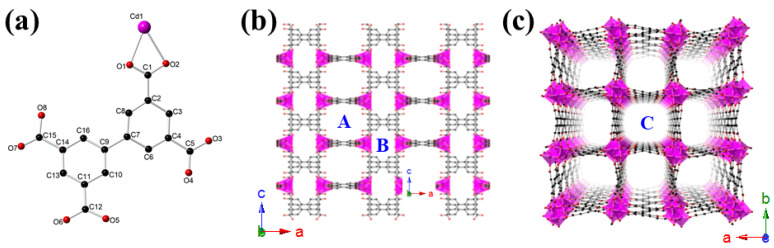
The asymmetric unit of CdBPTC (Cd, magenta; O, red; C, black) (**a**) and its polyhedral view of the 3D structure along b axis (**b**) and c axis (**c**).

**Figure 2:**
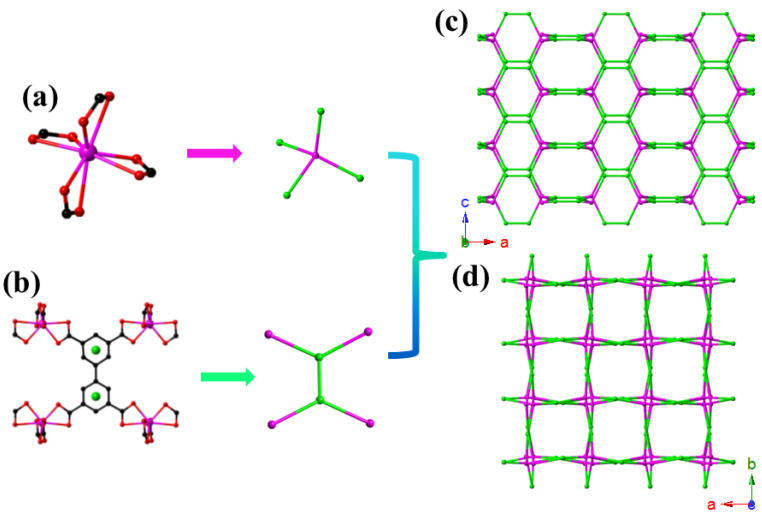
The simplified four-connected node of the mononuclear Cd^2+^ unit (**a**) and two 3-connected linkers (**b**). The (4, 3, 3)-connected topological along b axis (**c**) and c axis (**d**).

SXRD analyses revealed that CdBPTC crystallized in a tetragonal noncentrosymmetric *I*4*_1_cd* space group (No. 110). As shown in Figure 1a, the asymmetric unit of CdBPTC consists of a crystallographically independent Cd^2+^, a fully deprotonated BPTC^4−^ ligand. The coordination environment of the eight-coordinated Cd^2+^ center is composed of eight oxygen atoms from four chelating bidentate carboxylate groups, forming a simple mononuclear Cd(COO)_4_ SBU (Figure 2a). The BPTC^4−^ ligand serves as a μ_4_-bridge to link four Cd centers by a μ_4_-η^2^:η^2^:η^2^:η^2^ coordination mode through eight carboxylate oxygen atoms and extended into an infinite three-dimensional (3D) porous network (Figure 1b,c, Figure 2a, and Figure S1). CdBPTC owns three types of one-dimensional (1D) channel with dimensions of approximately 8.4 × 8.3 Å (pore A), 6.0 × 5.2 Å (pore B), and 9.7 × 8.4 Å (pore C), respectively, along the crystallographic a, b, and c axis, giving rise to the high porosity of 68.1% per unit cell volume, which is calculated by using the squeeze routine of PLATON [28]. Due to the presence of a large number of Me_2_NH_2_^+^ in the channel, hydrogen bonding forces may be generated with MOFs, resulting in the collapse of the frameworks after the removal of these ions. Unfortunately, we could not obtain specific surface area through conventional experiments. Crystal data and structure refinement parameters are summarized in Table 1 and Table S1.

The topological network pattern of CdBPTC is shown in Figure 2. In order to reflect the shape of the MOF channel, the BPTC^4-^ ligand and mononuclear Cd^2+^ unit were considered to be three- and four-connected nodes instead of four- and four-connected nodes, respectively. Topological analysis indicated that the structure could be simplified as a (4, 3, 3)-connected net with stoichiometry (3-c)_2_(4-c) and point symbol of {6^2^.8^4^}{6^2^.8}_2_. The phase purity of the as-synthesized CdBPTC was characterized by PXRD. As a result, the experimental peaks were consistent with the simulated one obtained by SXRD, indicating the samples were of high purity (Figure S2). However, there were slight differences in the low 2theta degrees due to the disorder of crystal structure after grinding (Figure S12).

### 2.2. Framework Stability

The TGA curve of CdBPTC revealed that the framework could be thermally stable up to ca. 360 °C (Figure S4). When the temperature rose to 140 °C, the weight loss was about 17.0%, which should be the escape of six water molecules. Then, the MOF slowly lost the two dimethylamine cations and began to collapse when the temperature exceeded 360 °C. The CdBPTC powders were immersed in different organic solvents such as DMF, DMSO, EtOH, etc. for 3 days, respectively. Then, the solid samples were filtered, followed by PXRD measurements. Comparing the PXRD patterns of the experimental and simulated samples, no obvious differences were found (Figure S5). These results demonstrated the excellent thermal and chemical stability of CdBPTC.

### 2.3. Luminescent and Sensing Properties

**Figure 3:**
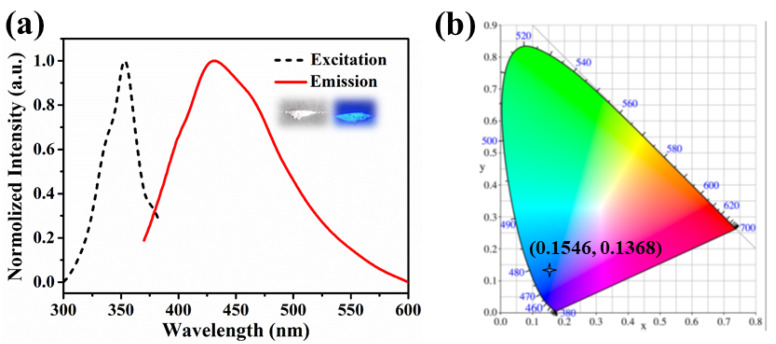
Excitation and emission spectra of CdBPTC, the insert shows samples under sunlight (left) and the ultraviolet light at 365 nm (right) (**a**), and the corresponding CIE chromaticity diagram showing the luminescence colors of CdBPTC (**b**).

As depicted in Figure 3, the luminescence characters of CdBPTC were performed in a solid state at room temperature. The sample showed blue fluorescence with an emission peak at 427 nm (excited at 354 nm), which could be mainly due to the intraligand charge transfer (ILCT) because the ligand H_4_BPTC also exhibited a similar emission (Figure S6).

#### 2.3.1. Fluorescence Detection of Metal Ions

**Figure 4:**
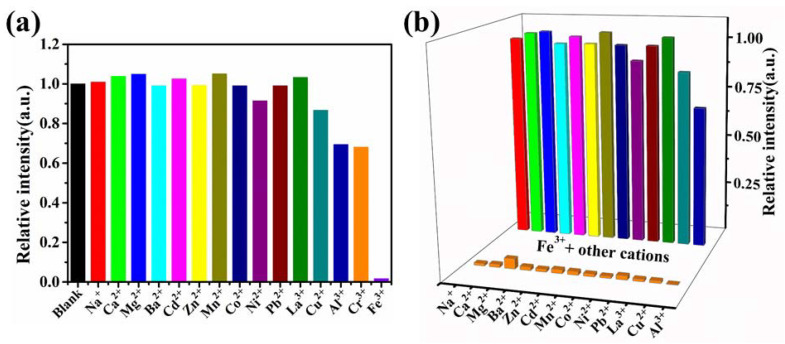
(**a**) The relative luminescence intensity of CdBPTC/DMF suspensions at room temperature after adding different metal ions, λex = 337 nm; (**b**) relative fluorescence intensity of CdBPTC + M^n+^ and CdBPTC + M^n+^ + Fe^3+^ at 427 nm (λex = 337 nm, M was Na^+^, Ca^2+^, Mg^2+^, Ba^2+^, Cd^2+^, Zn^2+^, Mn^2+^, Co^2+^, Ni^2+^, Pb^2+^, Cu^2+^, La^3+^, Al^3+^, and Fe^3+^, respectively).

A total of 15 mg ground CdBPTC sample was added to 15 mL DMF, then sonicated in an ultrasonic washer for 20 min; thus, suspended solutions were obtained. Na^+^, Ca^2+^, Mg^2+^, Ba^2+^, Cd^2+^, Zn^2+^, Mn^2+^, Co^2+^, Ni^2+^, Pb^2+^, Cu^2+^, La^3+^, Eu^3+^, Tb^3+^, Al^3+^, Cr^3+^, and Fe^3+^ aqueous solutions (30 μL 0.1 mol·L^−1^) were separately added to CdBPTC suspensions (3 mL), stirred, and tested the emission spectra immediately. Obviously, the fluorescence of MOF was almost completely quenched after the addition of Fe^3+^. In comparison, the addition of other cations did not cause an obvious fluorescence response, and only partial decreased luminescence intensity was observed for Al^3+^ and Cr^3+^ (about 30% lower than the original intensity; Figure 4a). After the addition of Eu^3+^ and Tb^3+^, the unique characteristic peaks of rare-earth ions were observed, but the emission peaks of MOF at 427 nm did not decrease significantly (Figures S14 and S15). CdBPTC exhibited suitable selectivity and responsiveness to Fe^3+^ with a turn-off effect.

Considering the influence of other ions on Fe^3+^ selectivity, 1.0 × 10^−3^ mol·L^−1^ Na^+^/Fe^3+^, Ca^2+^/Fe^3+^, Mg^2+^/Fe^3+^, Ba^2+^/Fe^3+^, Cd^2+^/Fe^3+^, Zn^2+^/Fe^3+^, Mn^2+^/Fe^3+^, Co^2+^/Fe^3+^, Ni^2+^/Fe^3+^, Pb^2+^/Fe^3+^, Cu^2+^/Fe^3+^, La^3+^/Fe^3+^, Cu^2+^/Fe^3+^, Al^3+^/Fe^3+^ competition experiments were also carried out. As shown in Figure 4b, the fluorescence intensity decreased significantly with the addition of the corresponding Fe^3+^. Additionally, the fluorescence quenching was completed immediately; that is to say, the turn-off effect still existed in the presence of other cations. The above results manifested that CdBPTC had high selectivity for Fe^3+^.

**Figure 5:**
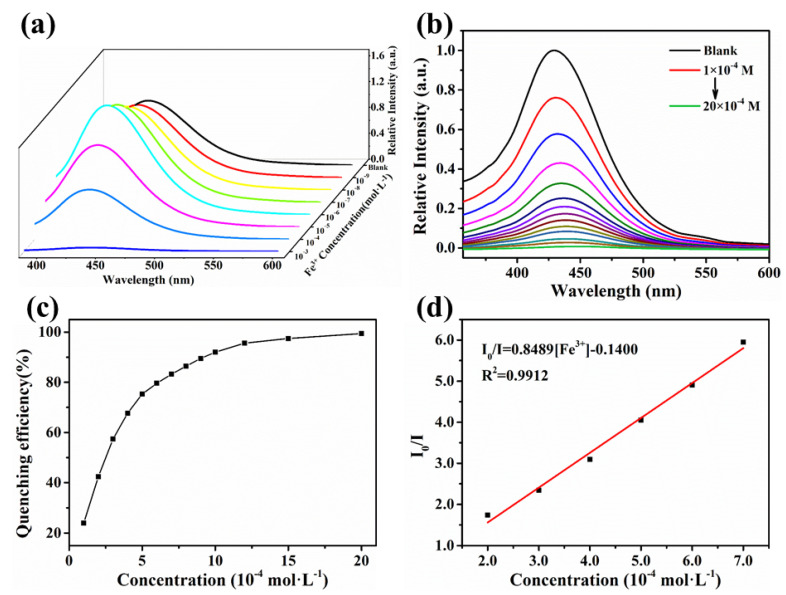
Liquid luminescence spectra of CdBPTC presenting turn-on (**a**) and turn-off (**b**) fluorescence signals with different concentrations of Fe^3+^ at room temperature, λex = 337 nm.; (**c**) quenching efficiency; (**d**) Stern–Volmer plot for the fluorescence intensity of CdBPTC after adding Fe^3+^.

To further study the sensitivity of CdBPTC for sensing Fe^3+^, the quantitative detection was investigated through the fluorescence titration spectra analyses. The fluorescence intensity of MOF gradually increased with the increase in Fe^3+^ concentrations in the range of 1.0 × 10^−9^~1.0 × 10^−6^ mol·L^−1^ (Figure 5a, the fluorescence measurements should be tested after 10 min with steady signals). In order to fairly evaluate the fluorescence enhancement effect of MOF in the presence of Fe^3+^ at very low concentrations, we conducted the same experiment on Cr^3+^, Tb^3+^, Eu^3+^, La^3+^, Zn^2+^, Mn^2+^, Fe^2+^ at the same concentration, respectively. As shown in Figures S13–S19, the fluorescence intensity also increased slightly in the presence of other metal ions. To eliminate the influence of a small amount of water in the solution, Fe^3+^ in DMF solution was selected for an experiment under the same conditions, and the same phenomenon was obtained, as shown in Figure S11. We speculate that this can be caused by the two Me_2_NH_2_^+^ contained in the MOF pore exchange ions with these metal cations. Interestingly, as the Fe^3+^ concentration reached 1.0 × 10^−4^ mol·L^−1^, the fluorescence intensity dropped sharply (Figure 5b). It is worth noting that attenuation of the fluorescence signals could be observed immediately. The fluorescence-quenching efficiency was calculated by using the formula: (1 − I/I_0_) × 100%, where I_0_ and I are the values of the luminescence intensity of pristine CdBPTC and Fe^3+^ loaded CdBPTC, respectively, indicating that CdBPTC showed a high sensitivity in sensing fluorescence quenching. The quenching efficiency was close to 100% when the concentration of Fe^3+^ reached 1.0 × 10^−3^ mol·L^−1^ (Figure 5c). Moreover, the luminescence-quenching coefficient (Ksv) can be quantitatively expressed by the linear Stern–Volmer equation [29,30] I_0_/I = 8489 [Fe^3+^] − 0.1400. The luminescent intensity at 427 nm presented a linear relationship at a concentration range of 2.0 × 10^−4^~7.0 × 10^−4^ mol·L^−^^1^ and R^2^ > 0.99 (Figure 5d). Therefore, the Ksv value could reach 8489 L·mol^−1^, which is comparable to the previously reported [31,32,33] better-performing materials. By the calculation of the 3σ/k formula (σ is the standard error; k is the slope), the detection limit for Fe^3+^ was found to be 9.04 ppm. In addition, we also tested the fluorescence spectra of CdBPTC in the presence of Fe^2+^ at different concentrations. As shown in Figure S19, the fluorescence intensity also showed obvious enhancement at 10^−9^~10^−6^ mol·L^−1^. When the concentration increased to 10^−3^ mol·L^−1^, the fluorescence quenched immediately, but the quenching efficiency (85%) was lower than that of Fe^3^^+^.

#### 2.3.2. Fluorescence-Sensing Mechanism of the CdBPTC Probe

According to previous reports, the luminescence effect of MOF caused by metal ions mainly results from the destruction of MOF structure, ion exchange, host–guest interaction, and iron quenched the fluorescence of MOFs, owning to the paramagnetic feature and significant visible absorbance of Fe^3+^. In order to investigate the possible mechanism, 15 mg CdBPTC solid sample was immersed into 1 mL 1.0 × 10^−2^ mol·L^−1^ DMF solutions of different metal ions (replace the metal salt solution after 24 h), respectively, and kept in dark for three days. Then, the solid fluorescence and PXRD were measured after filtration and drying (Figures S8–S10). The structure of CdBPTC before and after the addition of metal ions was studied. As shown in Figures S8 and S12, after adding metal ions, or after grinding MOF and then sensing Fe^3+^, or after soaking in water for 6 h, the framework was maintained, as confirmed by basically unchanged PXRD diffraction peaks, indicating that the luminescence quenching was not caused by the damage of the MOF structures.

Furthermore, given the anionic framework of CdBPTC, the increased fluorescence of MOF in the presence of metal cations at low concentrations may be the result of cation exchange. With the initial addition of Fe^3+^ (10^−9^~10^−6^ mol·L^−1^), the charge balance of the frameworks was gradually broken. After 10 min, the stable fluorescence signal indicated that the ion exchange between host and guest was complete, and the fluorescence intensity of MOF was enhanced. However, when the concentration of Fe^3+^ increased to 1.0 × 10^−4^ mol·L^−1^, the fluorescence quenching of MOF was obvious without waiting. To explore the possible mechanism, the UV–Vis spectrum of MOF in the solid state was tested (Figure S20). The absorption peaks of CdBPTC appear in the range of 200–350 nm. Moreover, the UV–Vis absorption spectra of Fe^3+^ with different concentrations were measured (Figure S21). No obvious absorption signals were observed at 10^−^^5^ and 10^−6^ mol·L^−1^. When the concentration reached 10^−4^ mol·L^−1^, significant absorption peaks began to appear in the range of 300~400 nm, which overlapped with the absorption wavelength of MOF. Meanwhile, the UV–Vis absorption spectra of Na^+^, Mg^2+^, Co^2+^, Ni^2+^, Pb^2+^, Cu^2+^, Mn^2+^, Zn^2+^, Fe^2+^, La^3+^, Al^3+^, Cr^3+^, Tb^3+^, and Eu^3+^ were measured at the same concentration, respectively, and only Fe^3+^ had an obvious absorption peak (Figure S21). In addition, the maximum emission wavelength of CdBPTC solid sample was located at 427 nm within the Fe^3+^ concentration in the range of 1.0 × 10^−9^~1.0 × 10^−6^ mol·L^−1^, while above 10^−3^ mol·L^−1^, a weak shoulder peak appeared at 470 nm in the solid-state fluorescence spectrum, whereas others showed little change (Figure S10). Therefore, we speculate that ion exchange is the main reason affecting the fluorescence of MOF at low concentration, while at high concentration, the competitive absorption of excitation source energy between metal ions and MOF leads to the fluorescence quenching of MOF [33,34,35,36]. Interestingly, when CdBPTC was exposed to Fe^3+^ in the DMF solution, the color of the solution changed from yellow to brown. By contrast, the naked eye recognition and detection for Fe^3+^ could be realized.

## 3. Materials and Methods

### 3.1. Chemicals and Reagents

All reagents and solvents including the H_4_BPTC ligand were purchased from commercial sources and used without further purification.

### 3.2. Apparatus

TGA was carried out in a nitrogen stream using the DTG-60 thermal analysis equipment with a heating rate of 10 °C·min^−1^ (Shimadzu, Kyoto, Japan). PXRD patterns of the bulk samples were measured on a Rigaku MiniFlex600 X-ray diffractometer (Rigaku, Kyoto, Japan). FTIR was obtained by KBr disks on a Shimadzu IRAffinity-1 FTIR spectrometer in the range of 4000–400 cm^−1^ (Shimadzu, Kyoto, Japan, abbreviations for the IR bands: w = weak, m = medium, b = broad, and vs. = very strong). Elemental analysis was carried out with Vario EL III CHNS equipment (Elementar, Langenselbold, Germany). The steady-state photoluminescence spectra (PL) for all samples were recorded on an RF-5301PC spectrofluorometer (Shimadzu, Kyoto, Japan).

### 3.3. Synthesis of {[Me_2_NH_2_]_2_[CdBPTC]·6H_2_O}n

A mixture of Cd(NO_3_)_2_·4H_2_O (0.05 mmol, 16 mg), 3,3′,5,5′-biphenyltetracarboxylic acid (H_4_BPTC, 0.0375 mmol, 13 mg), and mixed solvent *N*,*N*-dimethylformamide (DMF)/H_2_O (4.5 mL, 4:0.5, *v*/*v*), with an additive of drops of HNO_3_ (68%, 0.08 mL), was sealed in a Pyrex glass tube and heated in an oven at 90 °C for 72 h and cooled to room temperature at a rate of 5 °C·h^−1^. Colorless prism crystals were filtered and washed with DMF. Yield: ca. 90% based on H_4_BPTC. FTIR spectrum (KBr pellets, cm^−1^): 3442(br, m), 3078(br, m), 2789(m), 2472(w), 1616(s), 1562(s), 1435(s), 1413(s), 1363(s), 1315(m), 1018(w), 1014(w), 773(m), 731(m), 657(m), 651(m), 516(w), 420(w). Elemental analysis (CHN), (C_20_H_34_N_2_O_14_Cd)_n_, that is, {[Me_2_NH_2_]_2_ [Cd(BPTC)]∙6H_2_O)}_n_, calculated (%): C 37.60, H 5.36, N 4.38; found (%): C 37.72, H 5.29, N 4.67.

### 3.4. Determination of the Crystal Structures

A suitable size and high-quality crystal of CdBPTC was mounted at the end of glass fiber for single-crystal X-ray diffraction analysis. Data collection was performed by a Rigaku XtaLAB PRO MM007-DW diffractometer system, which was equipped with an RA-Micro7HF-MR-DW(Cu/Mo) X-ray generator and a Pilatus3R-200K-A detector (Cu Kα, *λ* = 1.54178 Å). The structure was solved by direct methods and refined by full-matrix least-squares refinements based on F^2^. Anisotropic thermal parameters were applied to all non-hydrogen atoms. The hydrogen atoms were generated geometrically. The crystallographic calculations were performed using the SHELXL programs [37,38]. The treatment for the guest molecules in the cavities of CdBPTC involves the use of the SQUEEZE program of PLATON. The existences of [Me_2_NH_2_]^+^ and H_2_O were further verified by the FTIR spectra, elemental analysis, and TGA data, obtaining the final chemical formulas of {[Me_2_NH_2_]_2_[CdBPTC]·6H_2_O}n.

### 3.5. Sensing Experiment

In metal ions sensing, 3 mg of CdBPTC was ground to a fine powder and ultrasonically dispersed into 3 mL DMF to prepare the 1 mg·mL^−1^ uniform suspensions. Various M(NO_3_)x aqueous solutions were added to the above suspensions. Then, the suspensions were loaded in the cuvettes for collecting the photoluminescence spectra. The fluorescence intensity of the suspension in DMF was measured as the blank sample.

## 4. Conclusions

In summary, we successfully synthesized an anionic microporous MOF, CdBPTC, with good thermal and chemical stability. CdBPTC was proved to be highly selective for the discrimination of ferric ions and showed excellent anti-inference ability toward many other cations. The luminescent intensity presented a linear relationship at a concentration range of 2.0 × 10^−4^~7.0 × 10^−4^ mol·L^−1^. The Ksv was about 8489 L·mol^−1^, which was obtained from the linear Stern–Volmer equation. This MOF exhibited a fluorescence “turn-off” effect for Fe^2+^ and Fe^3+^, which may be attributed to the competitive absorption of excitation source energy between ferric ions and CdBPTC. This type of MOFs can be developed as a potential candidate for the detection of Fe^3+^ in complex environments and can also be used for the determination of iron ion concentration in environmental hygiene, food safety, medical health, soil, and other media.

## Figures and Tables

**Table 1:** Crystal data and structure refinement results for CdBPTC.

Compound	CdBPTC
Empirical formula	C_16_H_6_CdO_8_
Formula weight	438.61
Temperature (K)	100.15
Crystal system	Tetragonal
Space group	*I*4_1_*cd*
*a*/Å	20.07520 (10)
*c*/Å	36.7826 (3)
Volume (Å^3^)	14,823.89 (19)
Z	16
*ρ*_calc_ (g·cm^−3^)	0.786
*μ* (mm^−1^)	4.897
*F* (000)	3424.0
Independent reflections	5869 [R_int_ = 0.0332]
Data/restraints/parameters	5869/25/215
Goodness of fit on F^2^	1.087
*R*_1_ [I ≥ 2σ(I)] ^a^	0.0399
wR_2_ [all data] ^b^	0.1365
Flack parameter	0.478 (16)
CCDC number	2,112,156

^a^ *R*_1_ = Σ(||*F*_0_| − |*F*_c_||)/Σ|*F*_0_|; ^b^ *wR*_2_ = [Σ*w*(*F*_0_^2^ − *F*_c_^2^)^2^/Σ*w*(*F*_0_^2^)^2^]^1/2^.

## Data Availability

The data presented in this study are available in supplementary material.

## Supplementary Materials

The following are available online. Figure S1: Ball-and-stick view of the coordination environments of Cd^2+^ and BPTC^4-^ in CdBPTC, Figure S2: PXRD patterns of CdBPTC, Figure S3: FTIR spectra of CdBPTC and H_4_BPTC ligand, Figure S4: TGA curve of CdBPTC, Figure S5: PXRD patterns of CdBPTC soaked in different organic solvents, Figure S6: The emission spectra of CdBPTC and H_4_BPTC ligand in solid state at room temperature, Figure S7: Photoluminescence spectra of CdBPTC in DMF and aqueous solutions containing different metal ions (1 × 10^−3^ mol·L^−1^) when excited at 337 nm, Figure S8: PXRD patterns of H_4_BPTC after being dispersed in DMF and different metal ions solutions for 72 h, Figure S9: Images of CdBPTC powder soaked in DMF solutions of different metal salts (1.0 × 10^−2^ mol·L^−1^) for 0 h (a) and 72 h (b), Figure S10: Solid emission spectra of CdBPTC after soaked in pure DMF, DMF solution of Fe^3+^ ion and other cations (1.0 × 10^−2^ mol·L^−1^) for 72 h. The inset shows the darker color of the CdBPTC sample after immersion in the solution of ferric ions, Figure S11: Liquid luminescence spectra of CdBPTC presenting turn-on and turn-off fluorescence signals with different concentrations of Fe^3+^ DMF solution at room temperature, λex = 337 nm, Figure S12: PXRD patterns of CdBPTC after grinding, sensing, or soaking in water, Figure S13: Liquid luminescence spectra of CdBPTC/DMF suspensions with different concentrations of Cr^3+^ at room temperature, λex = 337 nm, Figure S14: Liquid luminescence spectra of CdBPTC/DMF suspensions with different concentrations of Tb^3+^ at room temperature, λex = 337 nm, Figure S15: Liquid luminescence spectra of CdBPTC/DMF suspensions with different concentrations of Eu^3+^ at room temperature, λex = 337 nm, Figure S16: Liquid luminescence spectra of CdBPTC/DMF suspensions with different concentrations of La^3+^ at room temperature, λex = 337 nm, Figure S17: Liquid luminescence spectra of CdBPTC/DMF suspensions with different concentrations of Mn^2+^ at room temperature, λex = 337 nm, Figure S18: Liquid luminescence spectra of CdBPTC/DMF suspensions with different concentrations of Zn^2+^ at room temperature, λex = 337 nm, Figure S19: Liquid luminescence spectra of CdBPTC/DMF suspensions with different concentrations of Fe^2+^ at room temperature, λex = 337 nm, Figure S20: Solid UV–Vis spectra of CdBPTC and H_4_BPTC ligand, Figure S21: UV–Vis absorption spectra of Fe^3+^ aqueous solutions with different concentrations, Figure S22: UV–Vis absorption spectra of different metal cations in aqueous solutions (1 × 10^−3^ mol·L^−1^), Table S1: Selected bond lengths (Å) and bond angles (°) of CdBPTC.

## Sample Availability

Samples of the compounds are not available from the authors.
