# Supplementary material for: Construction of a Luminescent Cadmium-Based Metal–Organic Framework for Highly Selective Discrimination of Ferric Ions
Source: Molecules. 2021 Nov 13;26(22):6847. doi: 10.3390/molecules26226847 (PMC8625543; doi:10.3390/molecules26226847)
Supplement: Supplementary file 1 [file molecules-26-06847-s001.zip › molecules-1433344-supplementary.pdf]

molecules

MDPI

Supplementary Material

Construction of a Luminescent Cadmium-Based Metal–Organic Framework for Highly Selective Discrimination of Ferric Ions

Li-Li Xu [1,2], Qiu-Feng Zhang [1], Dong Wang [1], Guang-Wei Wu [1], and Hong Cai [1,2,*]

[1] School of Chemistry and Environmental Engineering, Hanshan Normal University, Chaozhou 521041, China; lilyncu@hstc.edu.cn (L.-L.X.); 2017137134@stu.hstc.edu.cn (Q.-F.Z.); wangd@hstc.edu.cn (D.W.); 2015136005@stu.hstc.edu.cn (G.-W.W.)

[2] Guangdong Provincial Key Laboratory of Functional Substances in Medicinal Edible Resources and Healthcare Products, Chaozhou 521041, China

* Correspondence: glcai@hstc.edu.cn

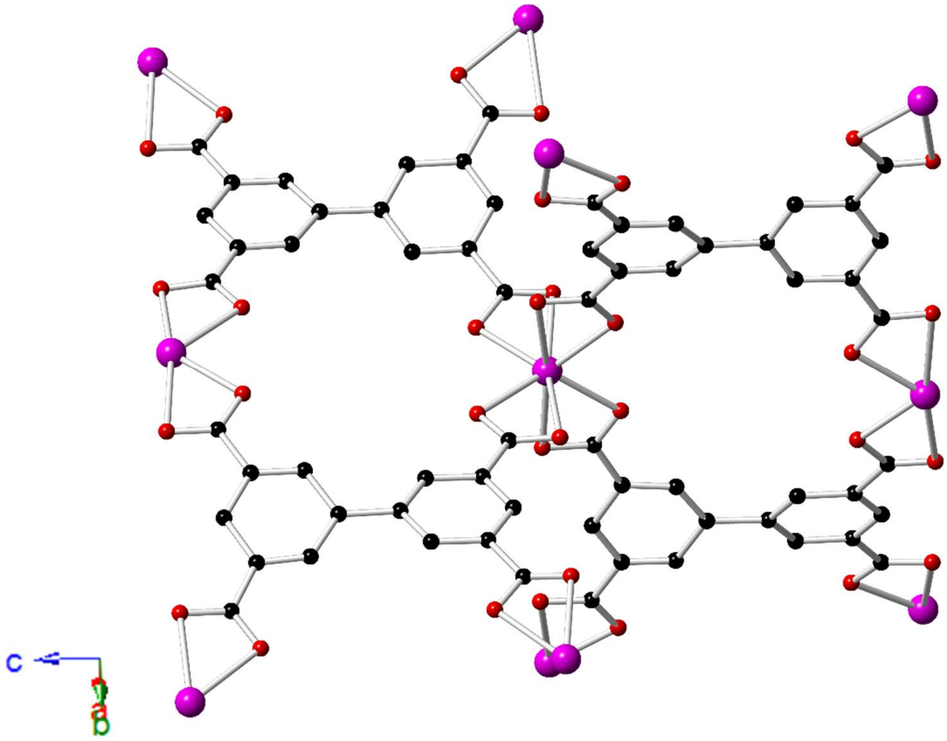

Figure S1. Ball-and-stick view of the coordination environments of Cd^{2+} and $BPTC^{4-}$ in CdBPTC.

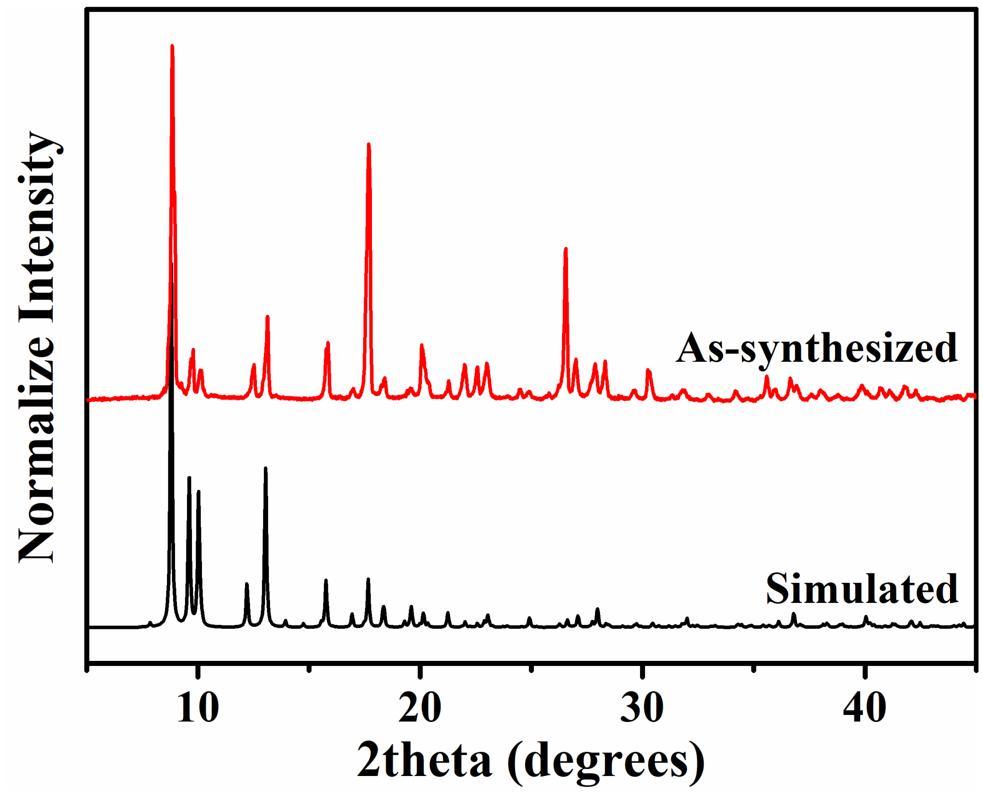

Figure S2. PXRD patterns of CdBPTC.

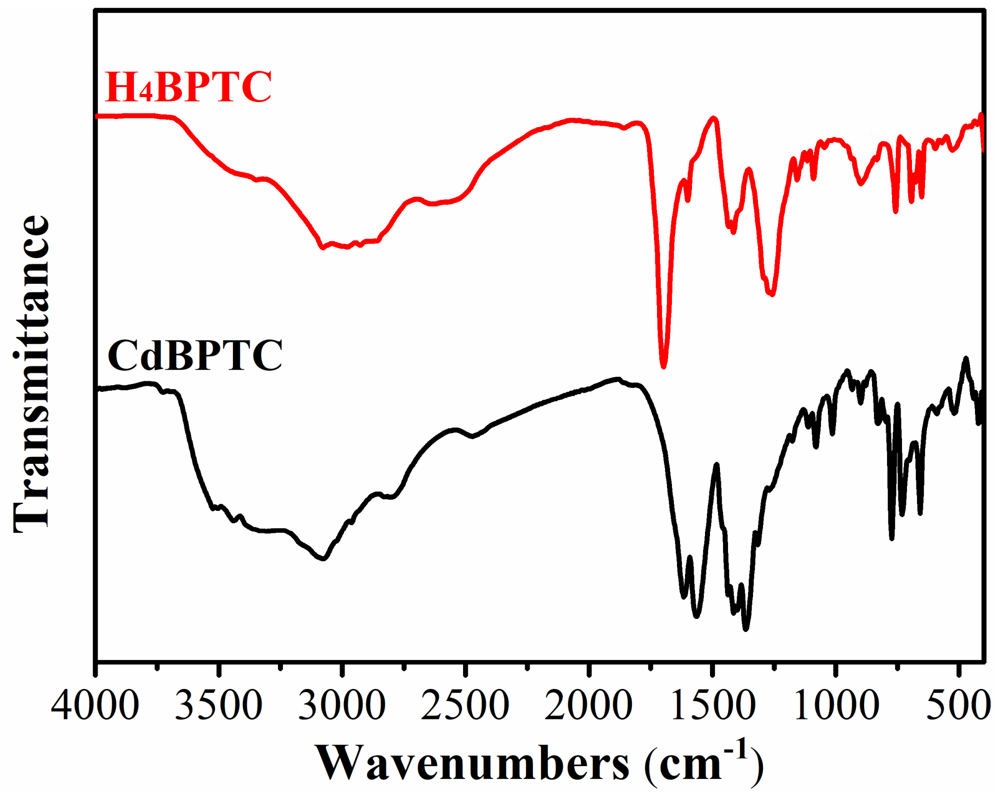

Figure S3. IR spectra of CdBPTC and H_4BPTC ligand.

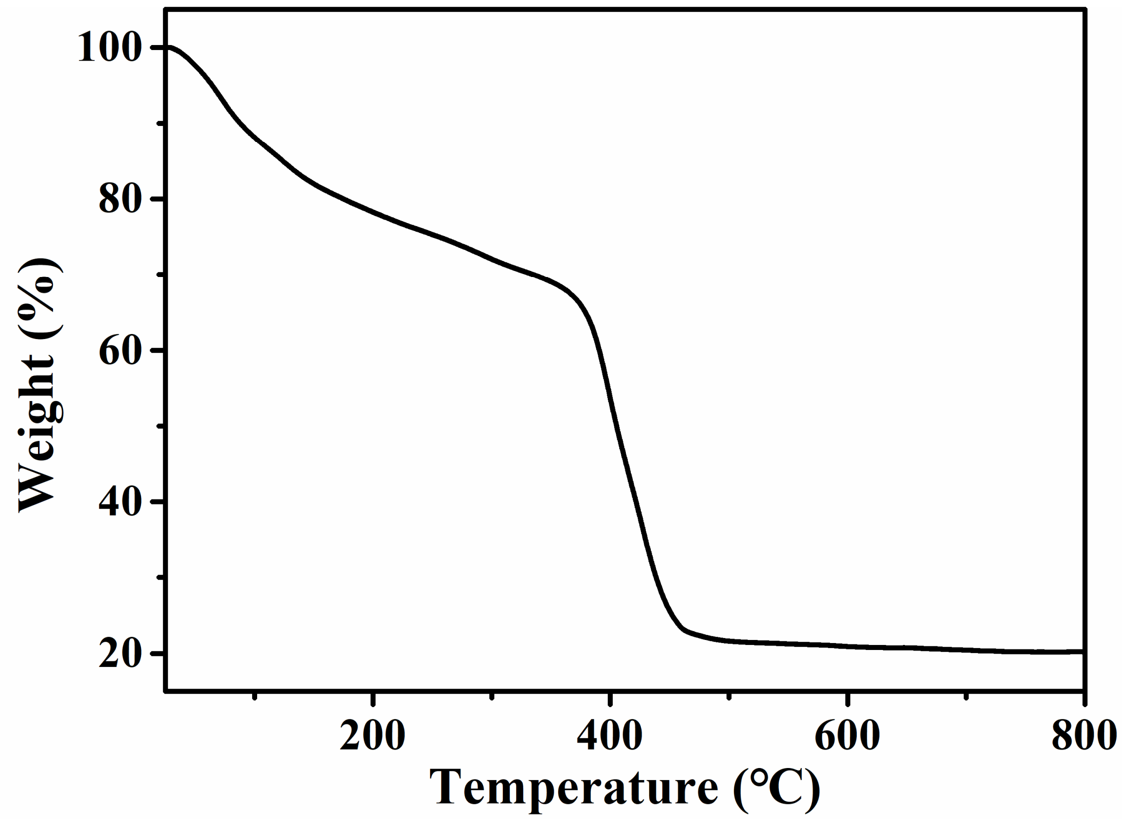

Figure S4. TGA curve of CdBPTC.

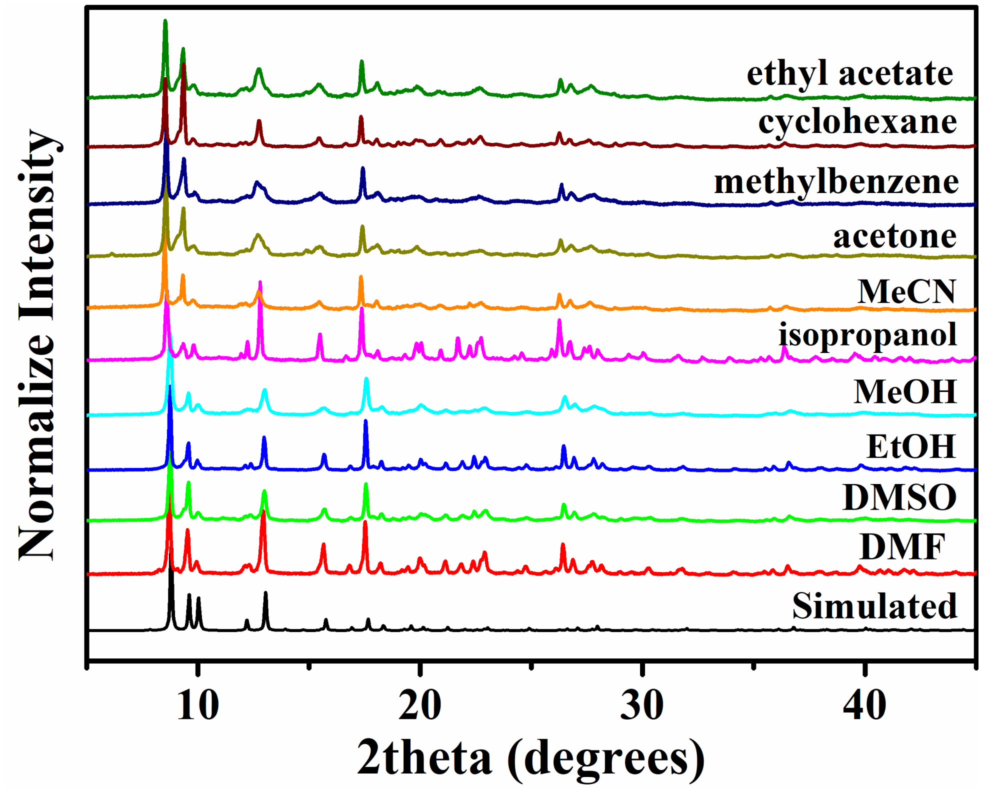

Figure S5. PXRD patterns of CdBPTC soaked in different organic solvents.

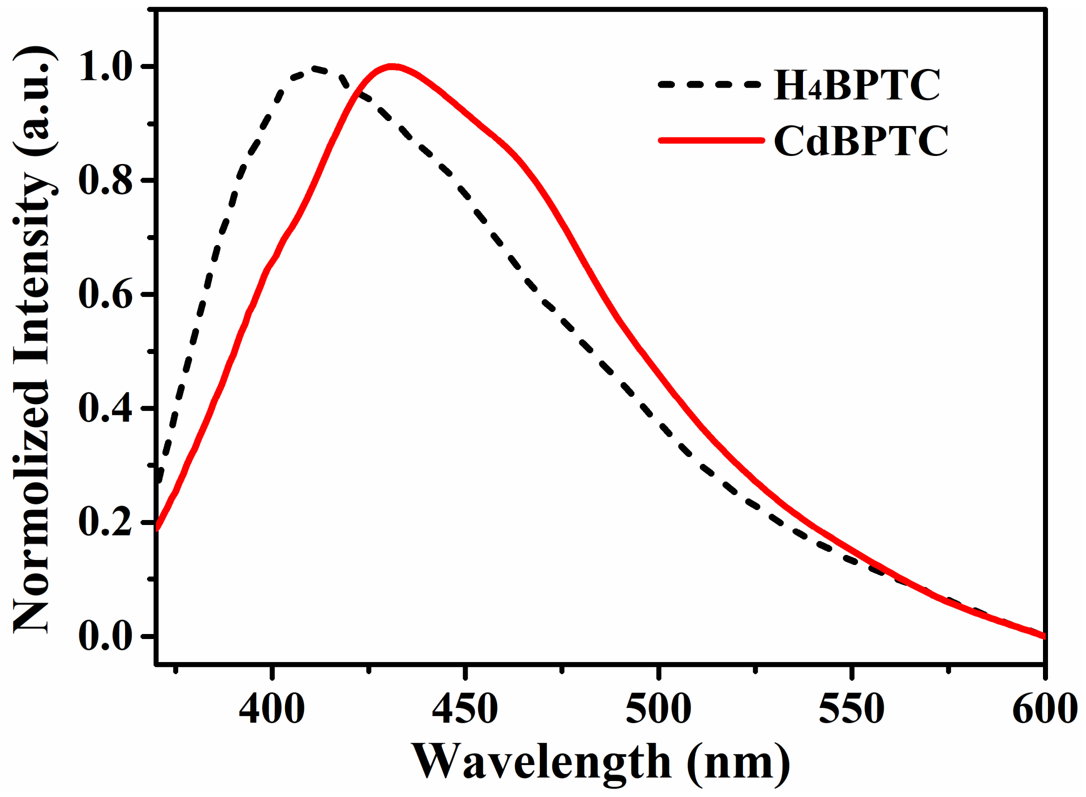

Figure S6. The emission spectra of CdBPTC and H_4BPTC ligand in solid state at room temperature.

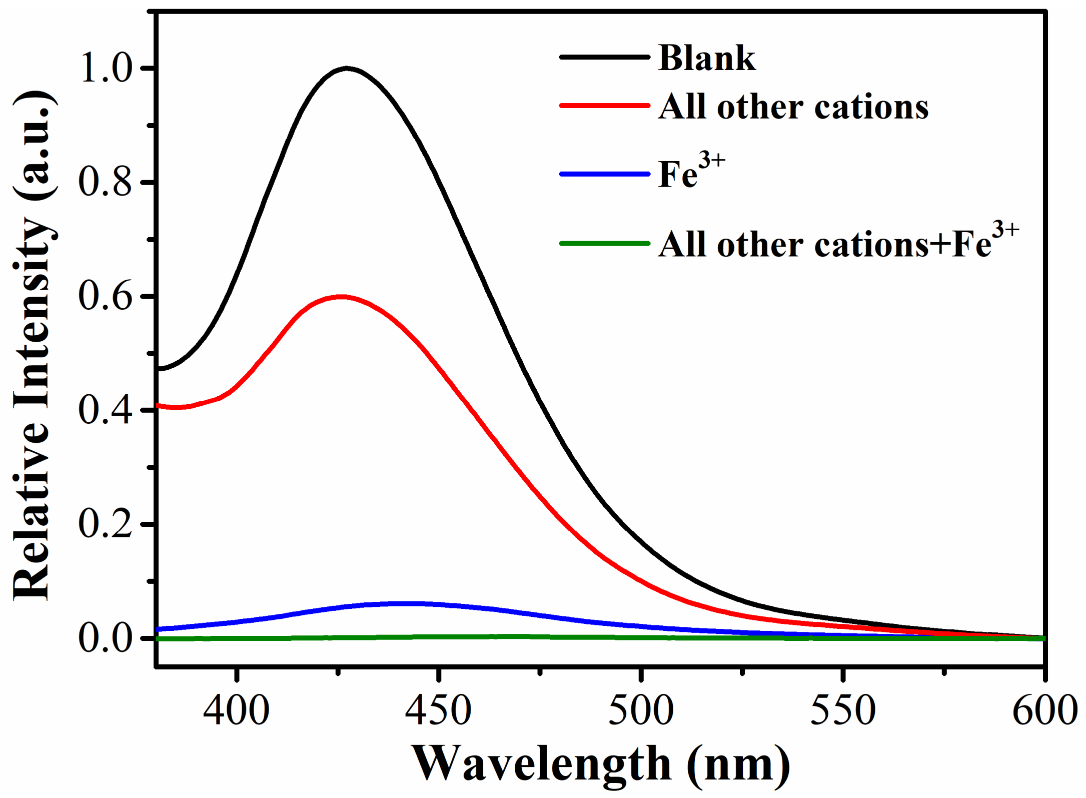

Figure S7. Photoluminescence spectra of CdBPTC in DMF and aqueous solution containing different metal ions (1.0×10^{-3} mol·L^{-1}) when excited at 337 nm.

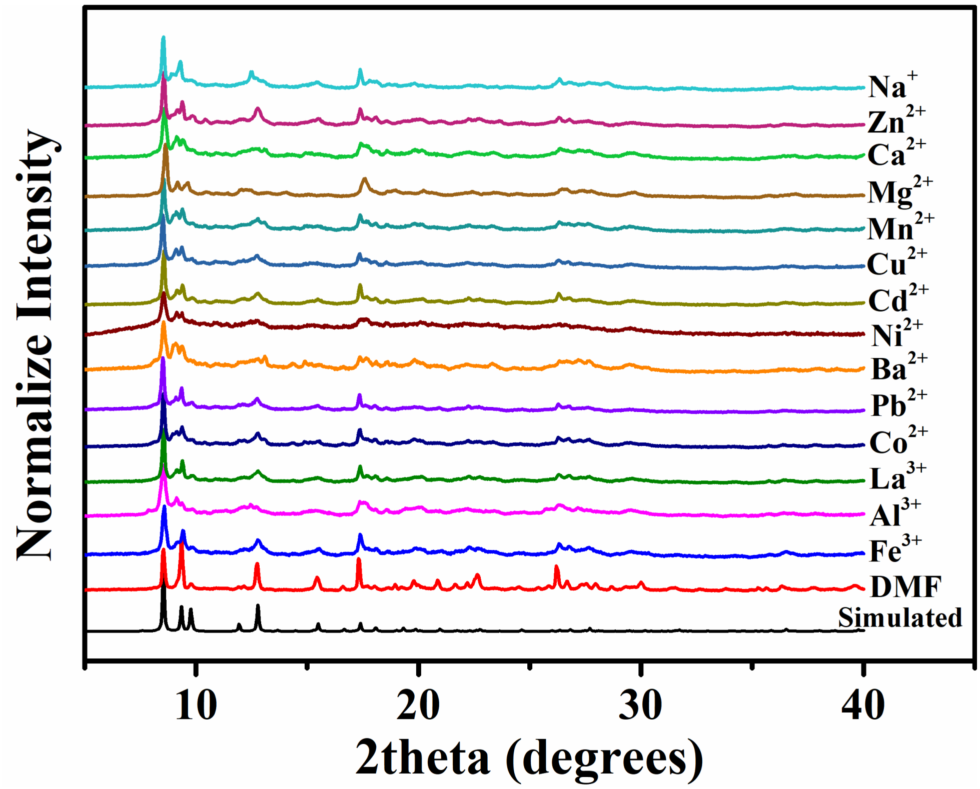

Figure S8. PXRD patterns of CdBPTC after being dispersed in DMF and different metal ions solutions for 72 hours.

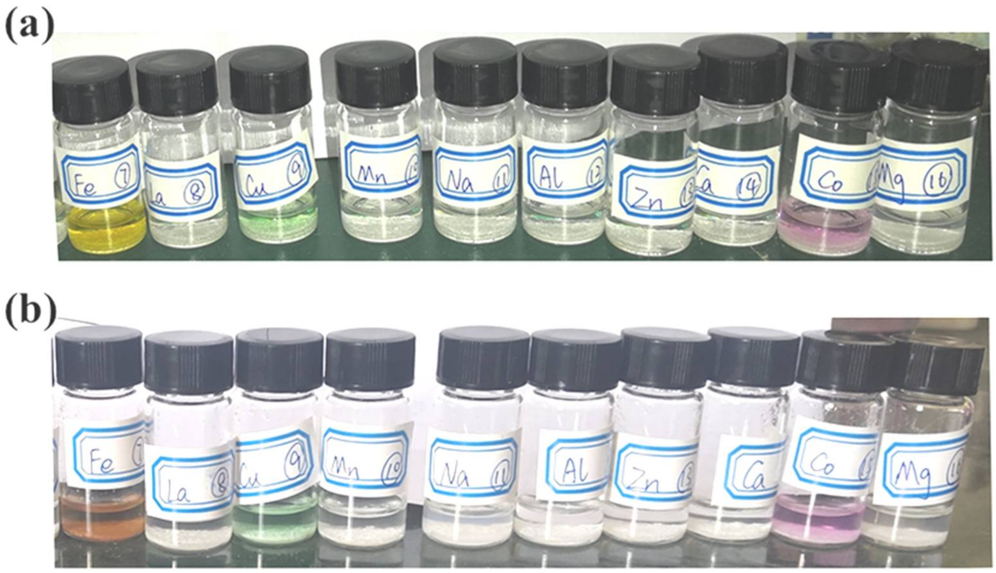

Figure S9. Images of CdBPTC powder soaked in DMF solutions of different metal salts (1.0 × 10^{-2} mol·L^{-1}) for 0 hours (a) and 72 hours (b).

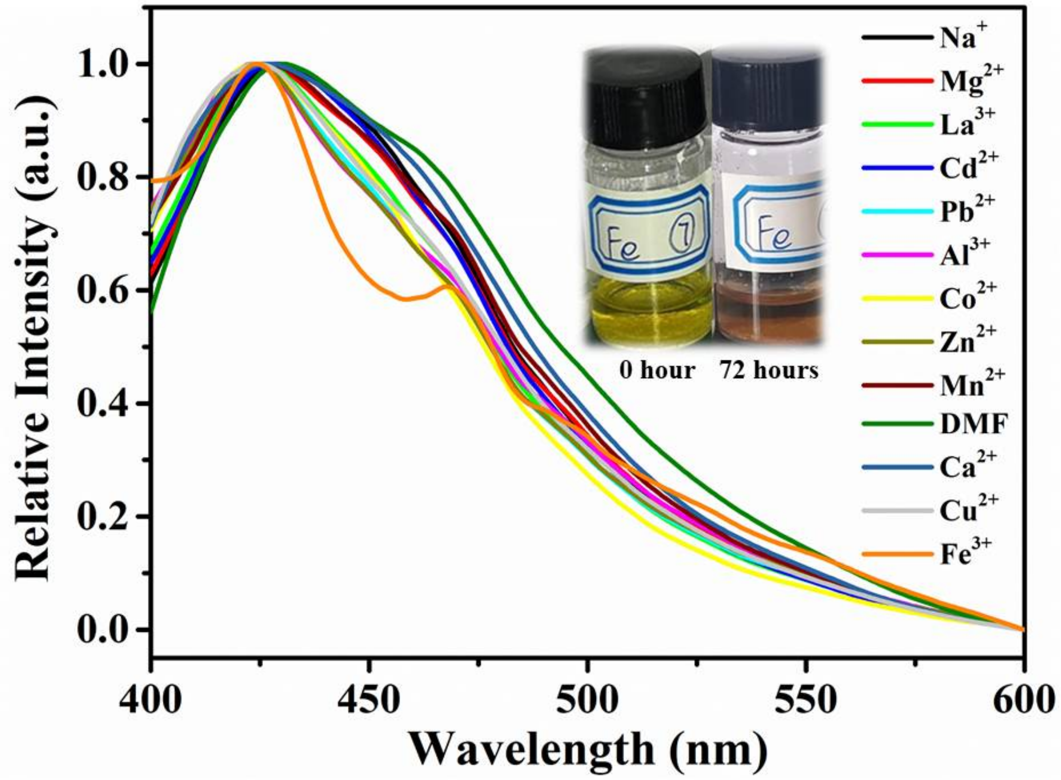

Figure S10. Solid emission spectra of CdBPTC after being soaked in pure DMF, DMF solution of Fe^{3+} ion, and other cations (1.0×10^{-2} mol·L^{-1}) for 72 hours. The inset shows the darker color of the CdBPTC sample after immersion in the solution of ferric ions.

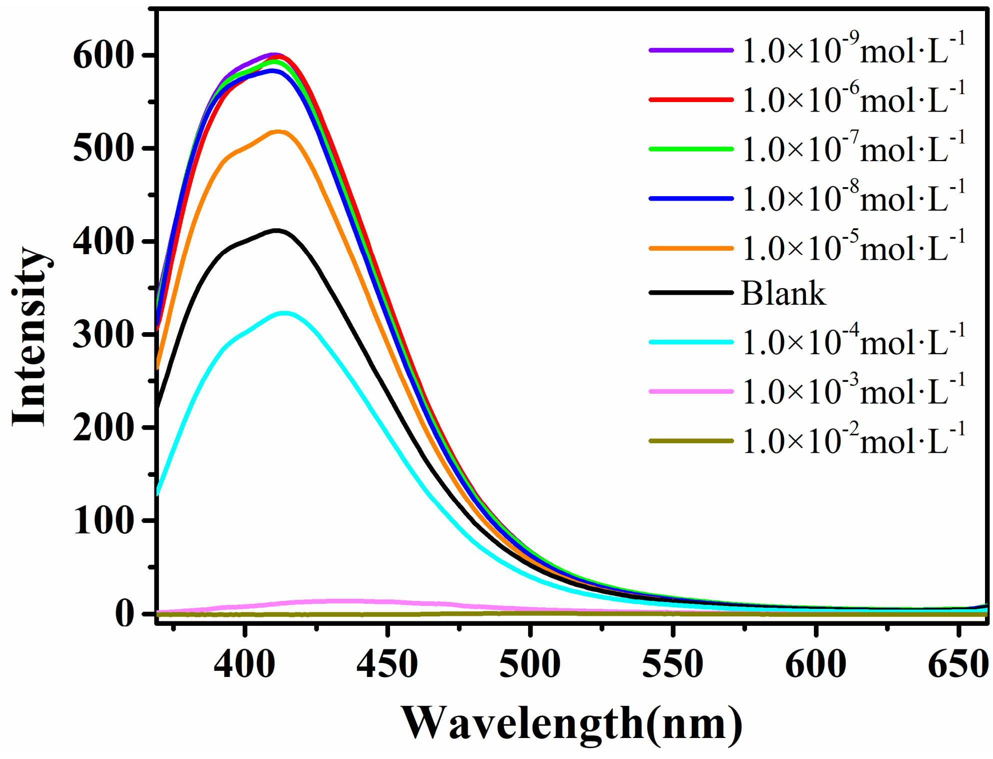

Figure S11. Liquid luminescence spectra of CdBPTC presenting turn on and turn off fluorescence signals with different concentrations of Fe^{3+} DMF solution at room temperature, λex = 337 nm.

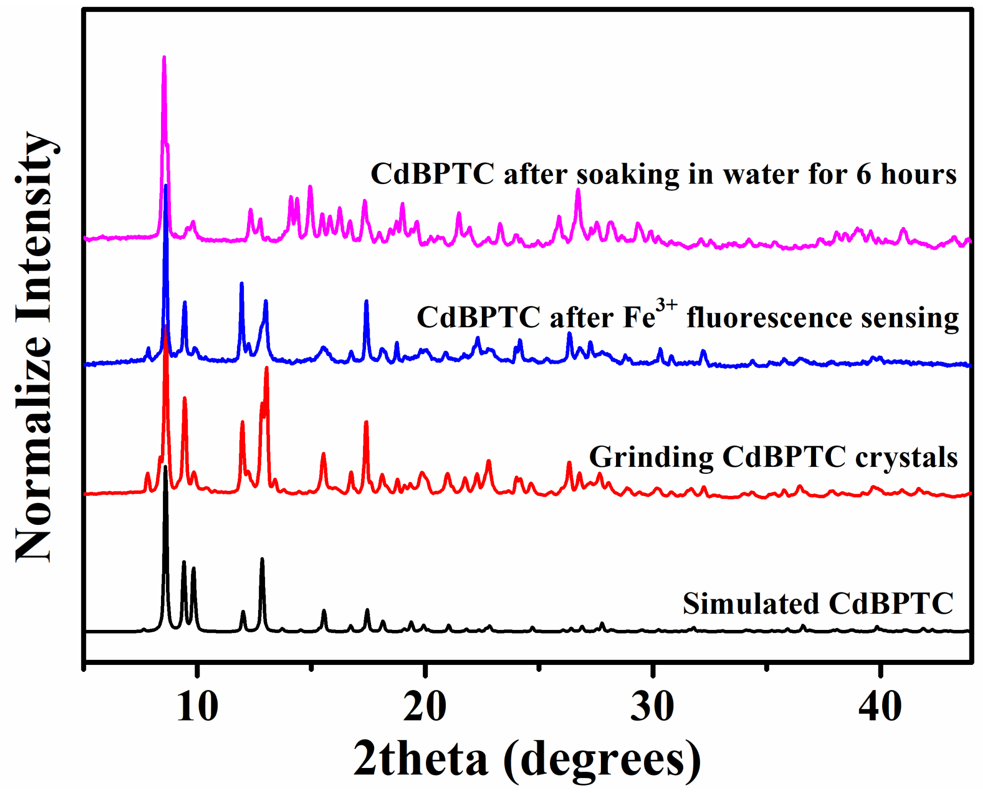

Figure S12. PXRD patterns of CdBPTC after grinding, sensing, or soaking in water.

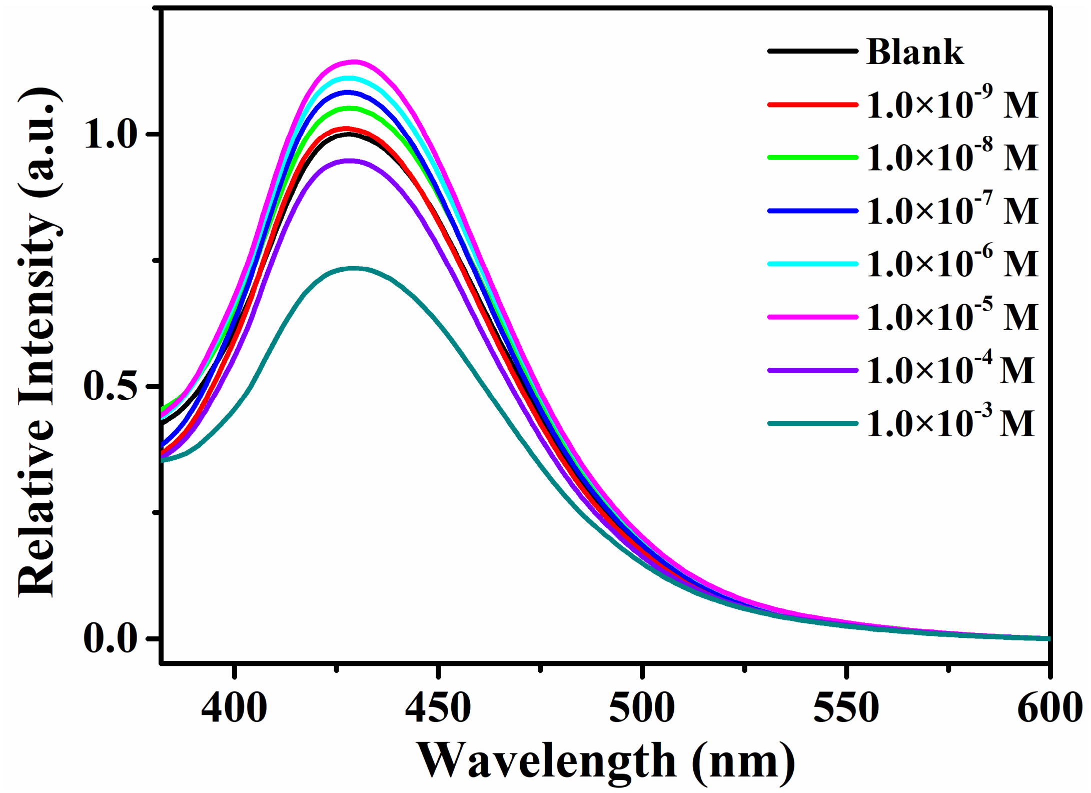

Figure S13. Liquid luminescence spectra of CdBPTC/DMF suspensions with different concentrations of Cr^{3+} at room temperature, λex = 337 nm.

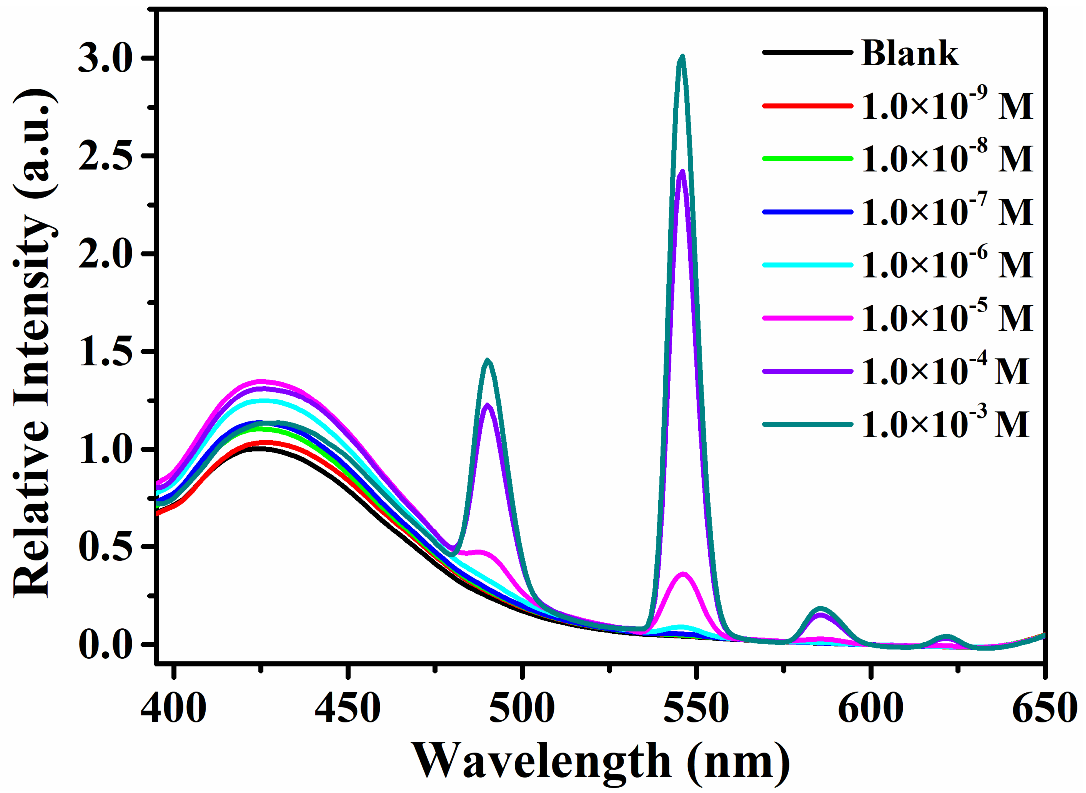

Figure S14. Liquid luminescence spectra of CdBPTC/DMF suspensions with different concentrations of Tb^{3+} at room temperature, λex = 337 nm.

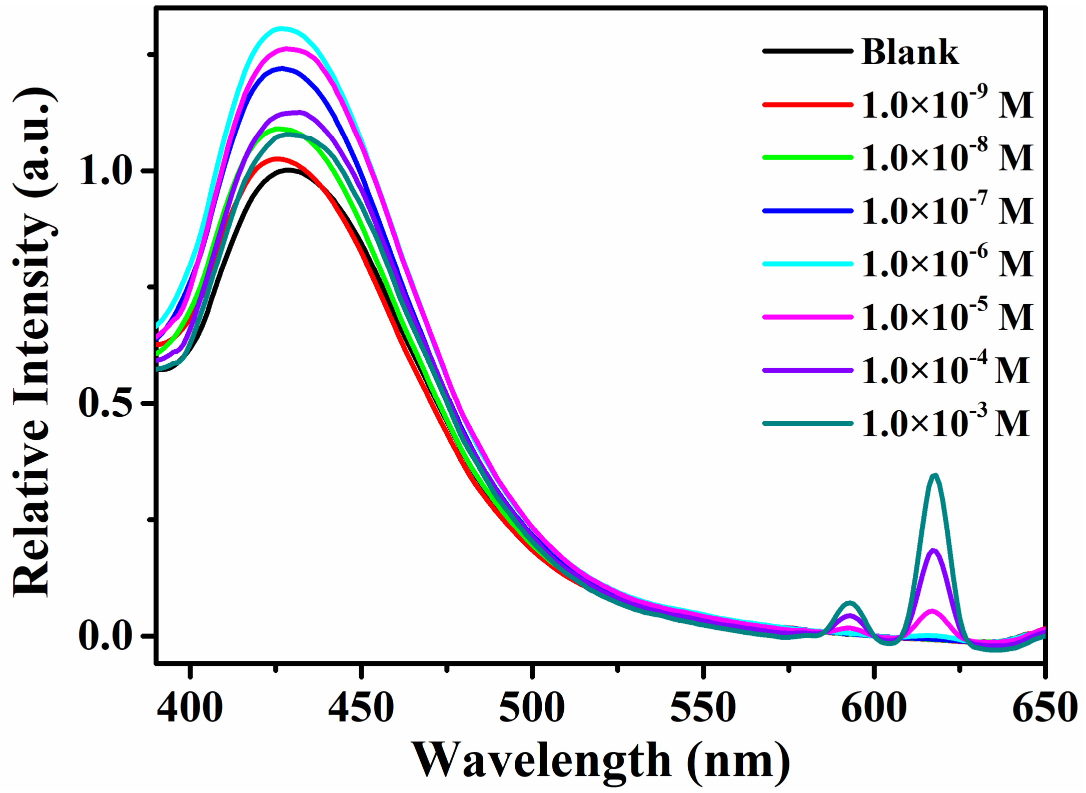

Figure S15. Liquid luminescence spectra of CdBPTC/DMF suspensions with different concentrations of Eu^{3+} at room temperature, λex = 337 nm.

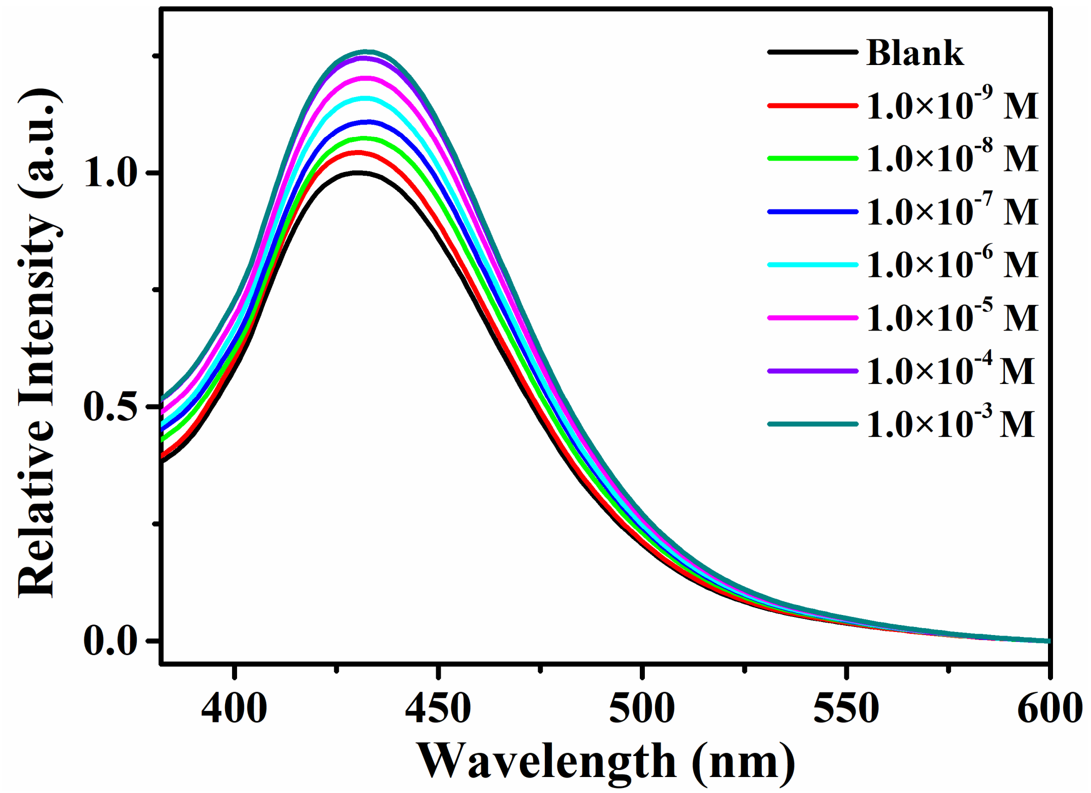

Figure S16. Liquid luminescence spectra of CdBPTC/DMF suspensions with different concentrations of La^{3+} at room temperature, λex = 337 nm.

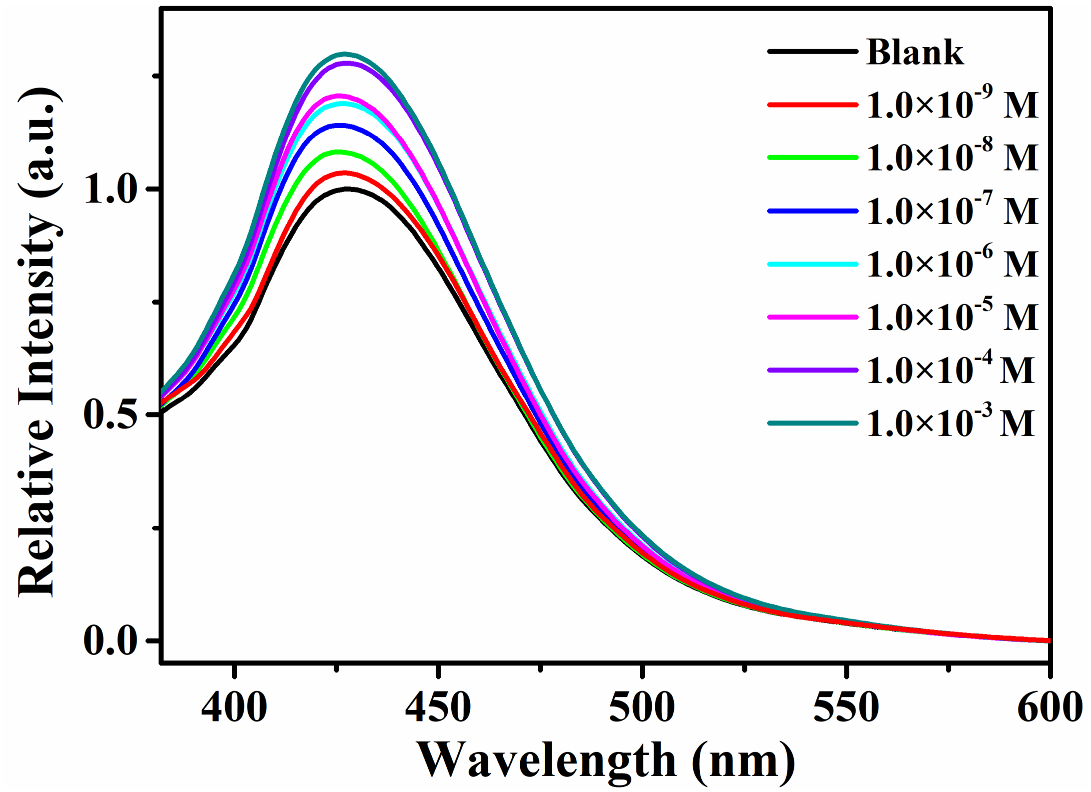

Figure S17. Liquid luminescence spectra of CdBPTC/DMF suspensions with different concentrations of Mn^{2+} at room temperature, λex = 337 nm.

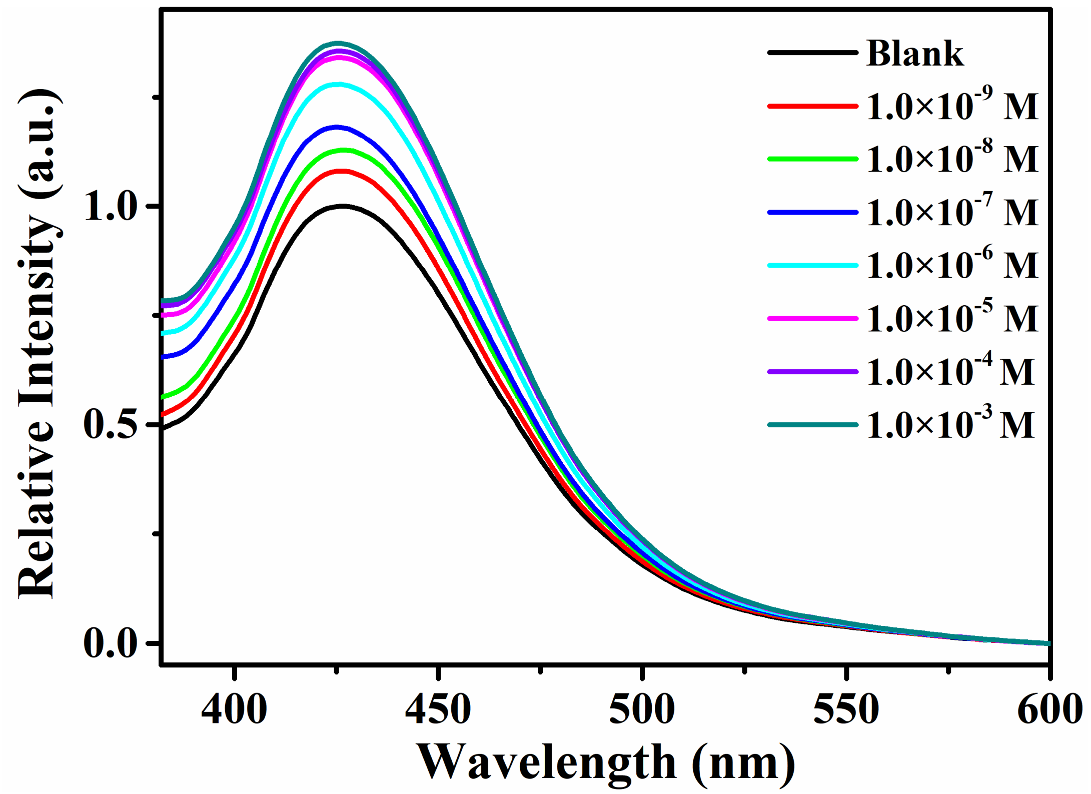

Figure S18. Liquid luminescence spectra of CdBPTC/DMF suspensions with different concentrations of Zn^{2+} at room temperature, λex = 337 nm.

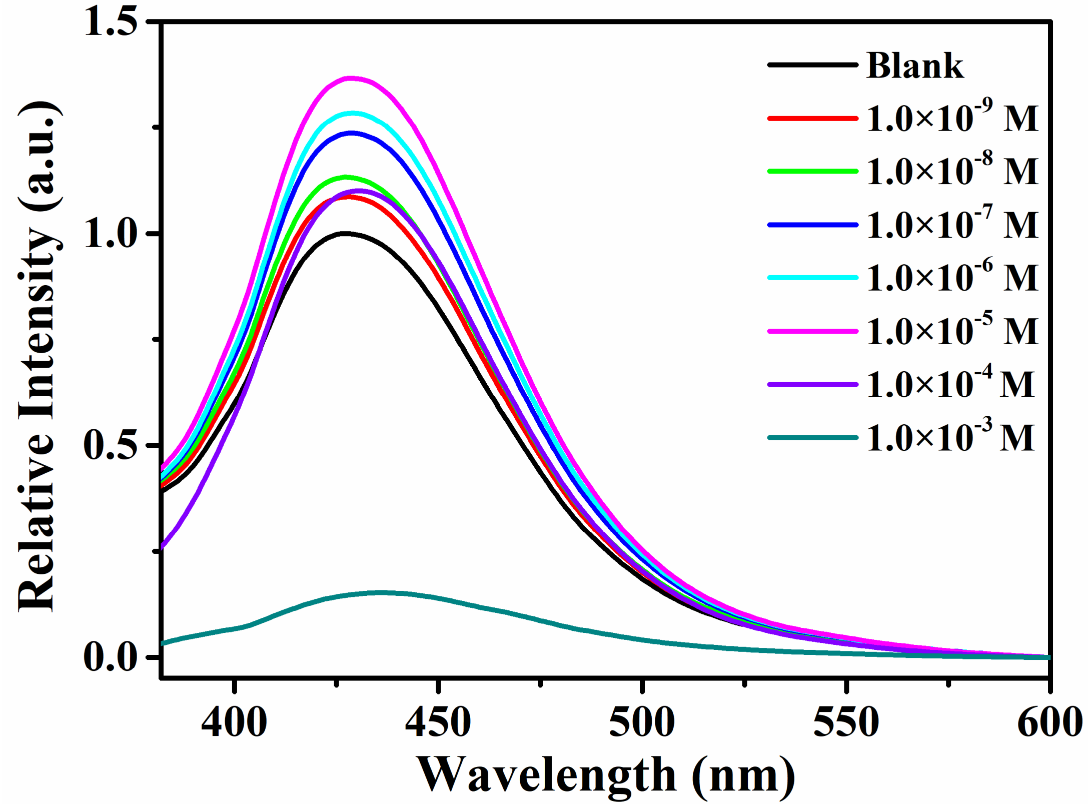

Figure S19. Liquid luminescence spectra of CdBPTC/DMF suspensions with different concentrations of Fe^{2+} at room temperature, λex = 337 nm.

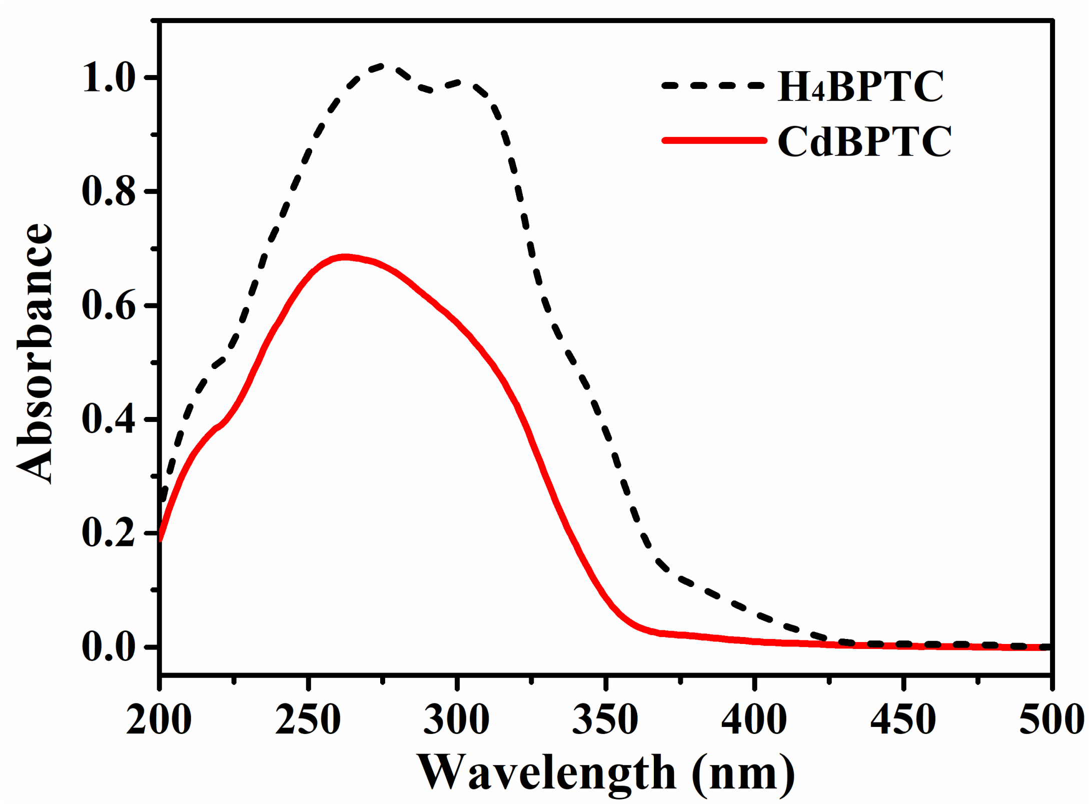

Figure S20. Solid UV–Vis spectra of CdBPTC and H_4BPTC ligand.

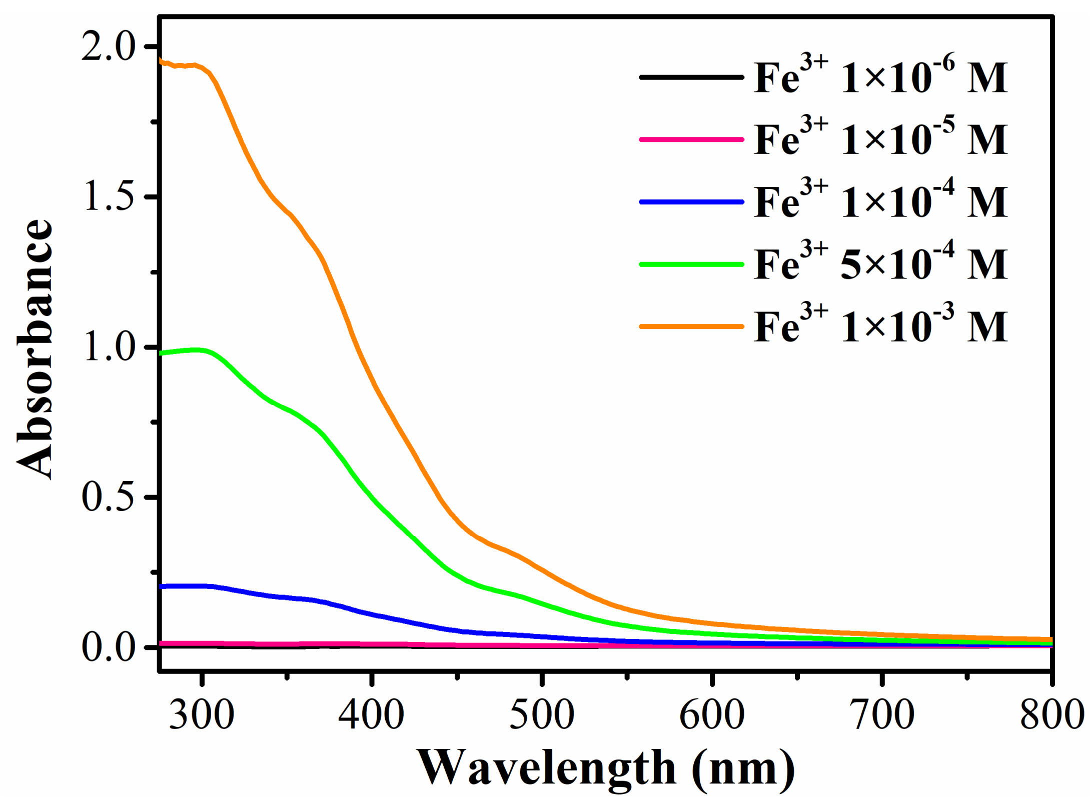

Figure S21. UV–Vis absorption spectra of Fe^{3+} aqueous solutions with different concentrations.

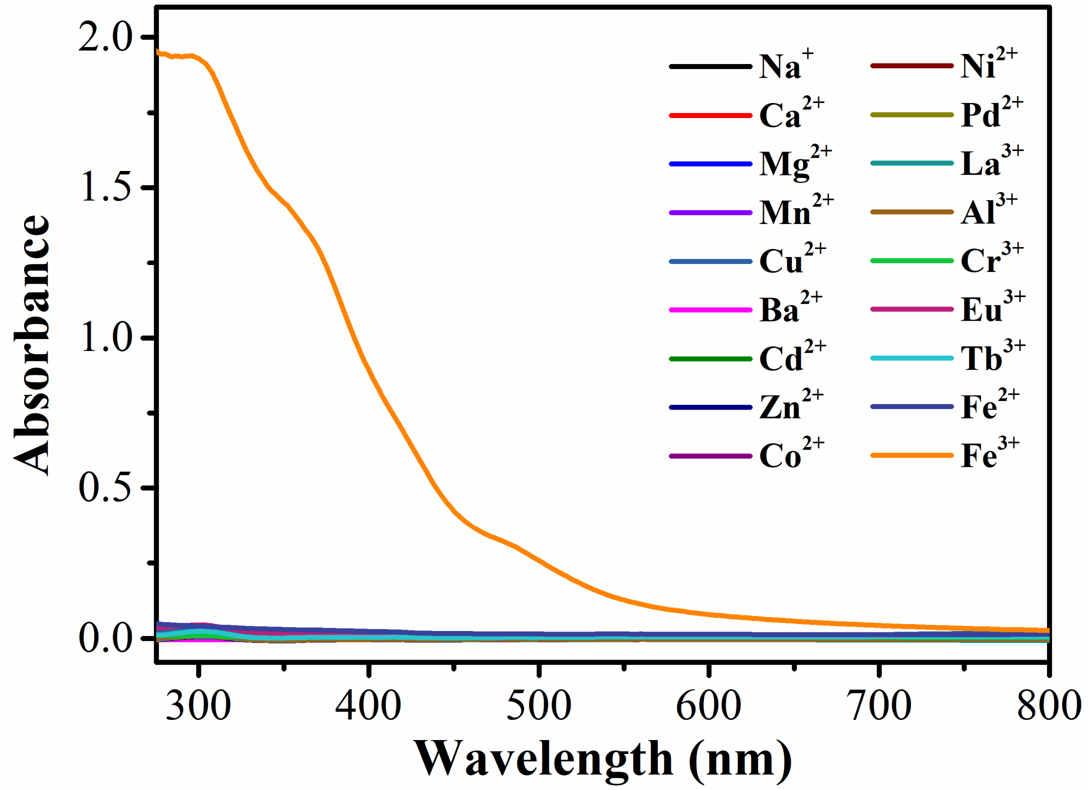

Figure S22. UV–Vis absorption spectra of different metal cations in aqueous solutions (1×10^{-3} mol·L^{-1}).

Table S1. Selected bond lengths (Å) and bond angles (°) of CdBPTC.

CdBPTC			
Cd1-O5^{1}	2.313(7)	Cd1-O6^{1}	2.592(6)
Cd1-O8^{2}	2.271(6)	Cd1-O3^{3}	2.590(5)
Cd1-O7^{2}	2.533(4)	Cd1-O1	2.330(7)
Cd1-O2	2.461(4)	Cd1-O4^{3}	2.290(7)
O5^{1}-Cd1-O7^{2}	135.1(2)	O2-Cd1-O6^{1}	90.76(19)
O5^{1}-Cd1-O2	86.1(2)	O2-Cd1-C15^{2}	98.12(17)
O5^{1}-Cd1-O6^{1}	54.4(2)	O2-Cd1-O3^{3}	168.0(2)
O5^{1}-Cd1-C15^{2}	110.1(3)	O6^{1}-Cd1-C15^{2}	161.7(2)
O5^{1}-Cd1-O3^{3}	81.99(19)	O3^{3}-Cd1-O6^{1}	81.39(18)
O5^{1}-Cd1-O1	127.64(19)	O3^{3}-Cd1-C15^{2}	87.09(16)
O8^{2}-Cd1-O5^{1}	82.6(3)	O1-Cd1-O7^{2}	88.35(17)
O8^{2}-Cd1-O7^{2}	52.87(19)	O1-Cd1-O2	53.8(2)
O8^{2}-Cd1-O2	92.34(19)	O1-Cd1-O6^{1}	90.2(2)
O8^{2}-Cd1-O6^{1}	136.6(2)	O1-Cd1-C15^{2}	107.9(2)
O8^{2}-Cd1-C15^{2}	27.8(2)	O1-Cd1-O3^{3}	134.7(2)
O8^{2}-Cd1-O3^{3}	87.27(17)	O4^{3}-Cd1-O5^{1}	123.41(17)
O8^{2}-Cd1-O1	125.44(18)	O4^{3}-Cd1-O7^{2}	82.08(19)
O8^{2}-Cd1-O4^{3}	120.7(2)	O4^{3}-Cd1-O2	136.1(3)
O7^{2}-Cd1-O6^{1}	166.1(2)	O4^{3}-Cd1-O3^{3}	52.4(3)
O7^{2}-Cd1-C15^{2}	25.1(2)	O4^{3}-Cd1-O6^{1}	84.04(19)
O7^{2}-Cd1-O3^{3}	89.82(15)	O4^{3}-Cd1- C15^{2}	100.1(2)
O4^{3}-Cd1-O1	82.6(3)	O2-Cd1-O7^{2}	99.51(17)

[1] 1/2-Y,1-X,1/4+Z; [2] +Y,1/2-X,1/4+Z; [3] -1/2+X,1/2-Y, +Z.
